# Beyond Mean Score Differences: Home Language and the Interpretation of Cognitive Test Performance in China

**DOI:** 10.3390/jintelligence14100251

**Published:** 2026-10-10

**Authors:** Man Xing, Jinkai Zhang, Gina S. Rhee

**Affiliations:** 1 College of Literature and Communication, Minzu Normal University of Xingyi, Xingyi 562400, China; manxing@xynun.edu.cn (M.X.); kelvinsoul@naver.com (J.Z.); 2 The Catholic College, The Catholic University of Korea, Bucheon 14662, Republic of Korea

**Keywords:** cognitive assessment, Mandarin oral character reading, home language, score interpretation, measurement validity, equivalence testing, overlap, CFPS

## Abstract

Large-scale surveys often use cognitive test total scores to describe group differences, but the construct meaning of such scores depends on task content, administration, language experience, and opportunities to learn. The China Family Panel Studies (CFPS) word test is an oral Mandarin character reading task with education-dependent entry and stopping rules. We analyzed 16,689 adults followed from 2010 to 2018 using the full survey strata, primary sampling units, and longitudinal weights, and contrasted baseline-adjusted, gain score, geographic overlap, propensity overlap, floor exclusion, nonlinear, and measurement error sensitivity results. The fully adjusted 2018 contrast relative to Mandarin-speaking homes was −0.023 SD (95% CI [−0.077, 0.030]) for Sinitic-dialect homes and −0.292 SD (95% CI [−0.430, −0.154]) for the pooled minority-language response category. The latter estimate was reduced to −0.183 SD after exact ethnicity code fixed effects and to −0.081 SD (95% CI [−0.291, 0.129]) under overlap weighting; only 49 respondents fell in the 1st–99th percentile propensity score intersection. A covariate-adjusted gain score model yielded a smaller minority-language contrast (−0.180 SD, 95% CI [−0.356, −0.005]). Correcting the baseline under plausible reliability values of 0.85–0.95 produced point estimates from −0.226 to −0.277 SD. Floor exclusions reduced but did not eliminate the negative contrast. These results show that the pooled minority-language coefficient is strongly estimand- and support-dependent. They support a constrained interpretation of the observed CFPS score rather than a direct inference about broad crystallized intelligence or a uniform effect of minority-language use.

## 1. Introduction

Cognitive tests produce performance scores under specific task and administration conditions. Moving from an observed score to a psychological construct requires a testable validity argument that addresses content coverage, administration procedures, examinee characteristics, intended score use, and plausible alternative explanations (Messick, 1995; Kane, 2013; American Educational Research Association et al., 2014). This requirement is especially important in comparisons across language groups. Character recognition depends on acquired knowledge, but it can also reflect exposure to the standard language, literacy experience, educational opportunity, comprehension of instructions, and familiarity with testing. Treating a total score difference as evidence of a general cognitive difference can therefore extend the construct claim beyond what the observed task supports.

China provides a useful setting in which to examine this problem. Mandarin functions as the principal language of schooling, public administration, and interregional communication, while Sinitic dialects and minority languages remain important in family interaction, local identity, and cultural transmission. Language experience is not categorical in everyday life: multilingual speakers differ in frequency of use, age of acquisition, literacy, code switching, and language choice across contexts. Contemporary bilingualism research accordingly warns against using a single group label as a substitute for the structure of language experience (DeLuca et al., 2019; Surrain & Luk, 2019; Navarro-Torres et al., 2021). Research on Mandarin dialect bidialectal adults similarly suggests that cognitive patterns depend on the demands of the task under study (Hsu, 2021).

The China Family Panel Studies (CFPS) repeatedly assesses individuals and families and includes both a word test and a mathematics test. The word task presents 34 Chinese characters in increasing difficulty and asks respondents to read them aloud in Mandarin. Administration is adaptive in a limited sense: education determines the entry point, and the sequence ends after three consecutive errors. The score therefore reflects more than visual recognition alone; it can depend on orthographic knowledge, lexical retrieval, standard Mandarin pronunciation, mapping between a home variety and Mandarin, literacy exposure, and speech production. The mathematics test is likewise closely linked to formal schooling (Huang et al., 2015). Both scores show systematic associations with age, education, urban–rural location, and birth cohort (Huang et al., 2015; Y. Qi & Xiong, 2023). These features make the CFPS well suited to a measurement question that is often obscured in large observational datasets: how much psychological interpretation can legitimately be attached to a language group difference in a survey test total score?

### 1.1. Language Background and Cognitive Test Comparability

The linguistic demands of cognitive tests have long been recognized as a source of validity concern in group comparisons. Earlier work showed that linguistic complexity and cultural loading can influence cognitive test performance (Cormier et al., 2014), while later evidence suggested that examinees’ own language characteristics may explain performance differences more strongly than the surface linguistic demands of test items (Cormier et al., 2022). The implication is that the same task can index a mixture of target ability, language exposure, and access to the test for respondents with different language histories. Contemporary intelligence assessment therefore places increasing emphasis on fairness, normative coverage, and the match between score interpretation and the population to which the score is applied (Holden & Tanenbaum, 2023; deLeyer-Tiarks et al., 2024).

Research on multilingual norms and measurement comparability reinforces this point. Reviews of neuropsychological instruments for Mandarin-speaking adults show that age, education, and language background need to be handled explicitly in score interpretation (W. G. Qi et al., 2022). Recent work further demonstrates that primary language use can affect the appropriateness of reference group norms and that monolingual norms cannot automatically be generalized to multilingual examinees (Kapoor et al., 2024; Ortiz & Cehelyk, 2024). Cross-language measurement invariance research likewise treats comparability across language versions as an empirical question rather than an assumption; for example, Grieder et al. (2025) formally tested the Dutch and German IDS-2 and found full invariance across language versions. Odermatt et al. (2026) similarly document the role of language factors in the assessment of cognitive and developmental functions. Group mean differences in observed scores therefore cannot be assumed to map directly onto differences in latent cognitive constructs.

### 1.2. Bilingual and Bidialectal Experience and Cognitive Performance

A second line of work concerns the heterogeneity of bilingual experience itself. DeLuca et al. (2019) conceptualized bilingualism as a spectrum of experiences and emphasized the importance of use intensity, context, and combinations of language experience. Navarro-Torres et al. (2021) similarly argued for a discovery science approach that reduces reliance on fixed group labels. Surrain and Luk’s (2019) systematic review showed substantial variability in how bilingualism has been defined and described across studies. For the present analysis, these findings imply that home language is necessarily a coarse ecological marker rather than a complete measure of proficiency, literacy, or degree of bilingualism.

Evidence concerning a general bilingual cognitive advantage also argues for restraint. Meta-analytic studies of adult executive functioning have not identified a stable overall advantage (Lehtonen et al., 2018), and systematic reviews of children have found effects that vary with task, sample, and design (Gunnerud et al., 2020). A population study of more than 11,000 adults likewise found no general executive function advantage associated with bilingualism (Nichols et al., 2020). Other work has nevertheless suggested benefits under particular life course conditions, including the possibility of cognitive reserve (Antoniou, 2019; Bialystok, 2021). Studies of Mandarin–Minnan and Mandarin–Hakka bidialectal adults have also reported task-dependent patterns (Hsu, 2021). Together, this literature favors task-specific and experience-specific interpretations over broad intelligence rankings derived from language categories.

### 1.3. Language, Schooling, and Cross-Domain Assessment

Mathematics is sometimes treated as a relatively low-language comparison domain, but mathematics tests are not language free. Abedi and Lord (2001) demonstrated the importance of language in mathematics assessment, and later multilingual assessment research showed that the language of testing can affect mathematics performance for some learners (Attar et al., 2022). Planas and Pimm (2024) likewise emphasized the roles of language, mathematical discourse, and multimodal communication in mathematics teaching and learning. Mathematics therefore cannot serve as a purely nonverbal benchmark. Its value here is comparative: it depends less directly on the pronunciation of Mandarin Chinese characters while still sharing formal schooling, test-taking experience, and symbolic learning with the CFPS word test.

This comparison is also compatible with an opportunity-to-learn perspective. Institutionally valued language practices are closely tied to schooling and socioeconomic opportunity (Bourdieu, 1991). In the present CFPS data, home language categories are also strongly patterned by education, region, and socioeconomic background. If similar language group differences appear in character recognition and mathematics, explanations based on shared educational opportunity and general test experience remain viable. If the difference is materially larger in character recognition, a task-specific linguistic requirement becomes more plausible. A cross-domain contrast can therefore function as a constraint on the set of admissible explanations rather than as a claim that mathematics is a language-neutral measure.

### 1.4. From Test Scores to Construct Interpretation

Cattell (1943) and Horn and Cattell (1966) distinguished fluid reasoning from knowledge accumulated through education and experience. The Cattell–Horn–Carroll (CHC) framework further places language development, lexical knowledge, and related acquired content within the broad comprehension knowledge domain (Schneider & McGrew, 2018; McGrew et al., 2023). That taxonomy, however, does not make every reading or vocabulary task an automatic measure of broad crystallized intelligence. Construct interpretation still requires adequate domain coverage, reliability, norms, administration comparability, and external validity evidence.

The CFPS word test requires respondents to read printed Chinese characters aloud in Mandarin, and educational attainment determines the initial item. Two validity risks follow. First, the task covers a narrower domain than broad crystallized intelligence, creating possible construct underrepresentation. Second, performance may also reflect Mandarin exposure, literacy opportunity, lexical access, pronunciation, dialect-to-Mandarin mapping, educational pathways, and test familiarity, creating possible construct-irrelevant variance for some intended interpretations. We therefore use the more literal label Mandarin oral character-reading performance. This label describes the observed task; it does not assume that the score is a pure measure of recognition, pronunciation, vocabulary, or broad crystallized intelligence.

The present study organizes score interpretation around four constraints. The first is a baseline constraint: matching domain performance in 2010 is used to distinguish pre-existing stratification from later conditional differences. The second is a domain constraint: standardized language group differences in character recognition and mathematics are compared, and equivalence tests distinguish failure to detect a domain difference from evidence that the difference is sufficiently small. The third is a model support constraint: common support restrictions and natural splines assess sensitivity to extrapolation and functional form. The fourth is a temporal constraint: balanced three-wave panels evaluate whether group trajectories remain separated, converge, or change rank over time. Together, these constraints determine how strong an interpretation can reasonably be attached to a total score difference.

A further issue arises when groups begin far apart on an error-prone baseline. ANCOVA and gain score analyses answer different questions and can diverge when baseline levels differ substantially, a problem classically illustrated by Lord’s paradox (Lord, 1967). In the present setting, the minority-language group begins more than 12 raw word score points below the Mandarin group. We therefore treat the baseline-adjusted model as one conditional estimand rather than as the unique longitudinal answer and compare it with change score and baseline measurement error sensitivity analyses.

### 1.5. The Present Study

Existing CFPS research has established that cognitive test scores are socially patterned, and that education, age, urban–rural residence, and historical cohort contribute to observed variation (Huang et al., 2015; Y. Qi & Xiong, 2023). What remains less clear is how language group differences in these survey scores should be interpreted once pre-existing observed performance, cross-domain patterns, model support, and time are considered jointly. The present study addresses that gap while separating an overall group difference from the narrower residual contrast targeted by the baseline-adjusted models.

**RQ1.**  *After conditioning on the observed 2010 word test score and the specified covariates, how large is the residual association between 2010 home language and 2018 Mandarin oral character-reading performance?*

**RQ2.**  *How do standardized home language contrasts in Mandarin oral character reading compare descriptively with those in mathematics, and do the cross-domain differences fall within the stated ±0.10 SD or ±0.15 SD equivalence regions?*

**RQ3.**  *How sensitive are the principal home language coefficients to geographic and multivariate overlap, baseline score floor restrictions, and nonlinear baseline specifications?*

**RQ4.**  *In a balanced 2010–2014–2018 panel, how do observed word test trajectories differ by home language, without interpreting wave-to-wave changes as change in an invariant latent construct?*

Education heterogeneity, self-rated Mandarin proficiency, short-term stability of reported home language, and panel attrition are treated as secondary or sensitivity analyses. The study was not preregistered. Accordingly, these additional specifications are interpreted as robustness and descriptive checks rather than as a preregistered confirmatory sequence, and they are not used to identify causal mechanisms.

## 2. Methods

### 2.1. Data and Analytic Samples

The CFPS is a multistage longitudinal survey of individuals, families, and communities in China (Xie & Hu, 2014; Xie & Lu, 2015). The sample flow begins with 33,598 adults interviewed in 2010. Among the 32,725 respondents in the three focal home language categories, 32,693 had a valid 2010 word score, 21,596 were linked to the 2018 person file, 16,729 had a valid 2018 word score and a positive longitudinal weight, and 16,689 had complete design/core covariates. The group-specific progression was Mandarin 6201 → 6195 → 3816 → 2922 → 2922 → 2918; Sinitic dialect 25,292 → 25,266 → 16,936 → 13,158 → 13,158 → 13,125; and pooled minority language 1232 → 1232 → 844 → 649 → 649 → 646, corresponding to final retention of 47.1%, 51.9%, and 52.4%, respectively. Thus, the smallest group was not disproportionately depleted relative to the other language categories. These retention rates alone do not rule out selective attrition. The complete cross-domain sample contains 16,686 respondents; the balanced three-wave word and mathematics panels contain 14,741 and 14,742 respondents, respectively. Table 1 summarizes the stage-specific sample flow by home language group. Counts in that table are unweighted, and percentages in parentheses represent retention from the 2010 focal language baseline within each language group. The two rows with *n* = 16,729 are identical because every respondent with a valid 2018 word score in the linked sample also had a positive 2018 longitudinal weight.

### 2.2. Measures

#### 2.2.1. Home Language

Home language is based on the 2010 CFPS item asking for the primary language used in daily communication with family members. Respondents selected one response: Mandarin Chinese, Chinese dialect, minority ethnic language, or other (Institute of Social Science Survey, Peking University, 2010). The main comparison retains the first three responses. The item records only this broad response category; it does not ask respondents who select “minority ethnic language” to identify which language they use. The CFPS questionnaire lists many examples in its definition of that response option, but those examples are not separate analytic categories in this item. Consequently, language-specific sample sizes, language-level disaggregation, and leave-one-language-out analyses cannot be recovered from this home language variable. Ethnicity is observed separately, but it is not used as a proxy for language identity.

The pooled minority-language response category is therefore an administrative survey category rather than a linguistically homogeneous population. It can include respondents differing in language family, script tradition, Mandarin exposure, ethnicity, region, migration history, and language of schooling. Its coefficient is best read as a weighted average conditional contrast among respondents who selected that broad CFPS response option. It is not an effect shared by specific minority languages and should not be generalized to all minority-language communities in China. More granular language analyses would require a variable that directly identifies the language used, which the present home language item does not provide.

#### 2.2.2. Cognitive Test Outcomes

The primary outcome is the comparable 2018 CFPS word test score (0–34), with the 2010 word test score serving as the matching-domain baseline. The administration is an oral reading task: the interviewer shows printed characters and asks the respondent to read them in Mandarin; scoring is based on the pronunciation response. The 2010 instructions explicitly state that tone, front-versus-back nasal distinctions, and flat-versus-retroflex tongue distinctions are not used to mark an answer wrong (Institute of Social Science Survey, Peking University, 2010). We therefore refer to the observed outcome as Mandarin oral character-reading performance. This wording does not imply a pure pronunciation measure: the response can also depend on orthographic recognition, lexical knowledge, literacy exposure, and mapping from a home language or dialect to standard Mandarin.

The word test contains 34 items ordered by difficulty. In the 2010 design, respondents with primary education or less began at item 1, those with junior–middle education began at item 9, and those with senior–high education or above began at item 21; testing stopped after three consecutive errors. The released composite score is the number of the hardest item answered correctly, and when no presented item is answered correctly the score is set to the item immediately below the education-based starting point (Institute of Social Science Survey, Peking University, 2010; Xu & Luo, 2012). Later administrations allowed for the interviewer to step down to an easier starting block after an initial failure, while the released variables wordtest14/wordtest18 and mathtest14/mathtest18 include versions intended to remain comparable with the 2010 fixed-start design (Institute of Social Science Survey, Peking University, 2022; Wu et al., 2021). The CFPS also used eight word forms and four mathematics forms, assigning different forms within households and across waves to reduce direct item carryover (Institute of Social Science Survey, Peking University, 2022). These design choices reduce some practice contamination but do not establish longitudinal measurement invariance or psychometric equivalence across education or language groups.

Published psychometric work on the 2010 CFPS word test provides some reassurance about score consistency but does not resolve the present measurement questions. Wu and Li (2016) reported reliability estimates of at least approximately 0.85 across the examined education/form groups, high correlations among alternative score constructions, and acceptable one-factor model fit. Those analyses concerned the 2010 literacy/word instrument. They did not establish comparable reliability or measurement invariance across the home language categories used here, or longitudinal invariance through 2018. Item-level differential item functioning (DIF) cannot be assessed using the total score analytic files available for the present study. Reliability below 1.00 also means that the 2010 baseline contains measurement error. Conditioning on the observed baseline therefore cannot be treated as conditioning on error-free latent proficiency.

Within each analytic sample, the 2018 outcome and its matching 2010 baseline are centered and standardized using the 2018 longitudinally weighted mean and standard deviation. Regression coefficients therefore represent conditional differences in outcome standard deviation units rather than fully standardized regression coefficients. The cross-domain analysis uses the comparable mathematics score (0–24) and its matching 2010 baseline. Standardizing the two outcomes places their group contrasts on a common SD scale, but it does not make the tests equivalent in reliability, difficulty, content, educational exposure, or linguistic demand. Item-level longitudinal invariance and formal equating across home language groups cannot be evaluated with the available total score analytic files; three-wave models are therefore interpreted only as patterns in observed CFPS scores.

#### 2.2.3. Covariates and Secondary Language Measures

Baseline covariates include age, age squared, sex, urban–rural residence, ethnicity, years of education, highest parental educational attainment, log per-capita household income, and province indicators. Missing parental education and income are replaced with the sample median and accompanied by missingness indicators. This is a pragmatic single imputation plus indicator strategy rather than a substitute for multiple imputation; residual sensitivity to the missing-data model remains possible. Years of education are centered at nine years. Self-rated Mandarin proficiency from 2012 ranges from 1 to 5 and is converted to a longitudinally weighted z score for the secondary model.

### 2.3. Survey-Weighted Models and the Conditional Estimand

Primary regressions use the 2018 national longitudinal weight and the CFPS multistage design, specifying the six 2010 sampling strata (subpopulation) and 176 PSUs. Model 1 includes home language, the observed 2010 word test baseline, age, age squared, and sex. Model 2 additionally includes urban–rural residence, ethnicity, education, parental education, household income, and missingness indicators. Model 3 further includes province indicators. The survey design has 170 design degrees of freedom; the residual design degrees of freedom are 164, 157, and 133 for Models 1–3, respectively. The target of Model 3 is deliberately narrow: the expected 2018 between-group difference among respondents with the same observed 2010 word test score and the same values of the measured covariates. Because conditioning on 2010 removes baseline differences that may themselves embody long-standing language exposure, schooling, and literacy opportunity, Model 3 is not an estimate of the overall association between home language and 2018 performance, a total language effect, or eight-year cognitive growth.

For the cross-domain analysis, each respondent contributes one word test record and one mathematics record, with repeated records nested within respondents and PSUs. The model allows for the matching baseline and all covariates to have domain-specific slopes. A joint domain by home language Wald test evaluates whether the two standardized group contrasts differ statistically, and group-specific domain differences are calculated as the word test contrast minus the mathematics contrast. This is a descriptive cross-domain comparison rather than identification of a language-specific test effect: the two tests differ in scale, content, educational exposure, difficulty, reliability, and linguistic requirements, and standardization does not remove those differences.

A nonsignificant domain by home language interaction does not establish practical equivalence. We therefore use two one-sided tests (TOST) with 90% confidence intervals to assess a researcher-chosen ±0.10 SD equivalence region and a ±0.15 SD sensitivity region (Lakens et al., 2018). These bounds operationalize small, standardized cross-domain differences for the present analysis; they are not externally validated CFPS, clinical, or causal cutoffs.

### 2.4. Geographic Identification, Multivariate Overlap, and Functional Form Sensitivity

We first retain the earlier marginal support checks based on the intersection of group-specific 1st–99th and 5th–95th percentile ranges for the observed 2010 standardized word score and years of education. Because these two margins cannot establish joint covariate support, we add two stronger diagnostics. First, province-by-language summaries identify where Mandarin and minority-language respondents coexist and quantify how much of the pooled category is represented in such provinces. We also re-estimate Model 3 within provinces containing at least one, and then at least five, respondents from both groups, and perform leave-one-province-out analyses. Second, for the minority-language versus Mandarin comparison, we fit a survey-weighted quasibinomial logistic model for minority-language home use using the original 2018 longitudinal weights, six 2010 sampling strata, and PSU clustering. Predictors are baseline word score, age, age squared, sex, urbanicity, ethnicity, education, parental education, income, the two missingness indicators, and province fixed effects. Let *e_i_* denote the fitted probability of minority-language home use and *w_i_* the 2018 longitudinal weight. For numerical stability *e_i_* is bounded to [10^−6^, 1 − 10^−6^]; no trimming or truncation of the resulting overlap weights is applied. The overlap weight is *w_i_*(1 − *e_i_*) for minority-language respondents and *w_i_ e_i_* for Mandarin respondents. We report the 1st–99th percentile propensity intersection, standardized mean differences before and after weighting, Kish effective sample sizes, a support-restricted model, and an overlap-weighted outcome model using the same survey strata and PSU structure. Kish effective sample size is calculated as (∑_i_ w_i_^*^)^2^ / ∑_i_ (w_i_^*^)^2^, where w_i_^*^ denotes the final overlap weight. These analyses diagnose where the original regression relies on extrapolation rather than treating regression adjustment itself as evidence of common support.

To reduce dependence on functional form, we additionally fit natural spline specifications for baseline word score and education. Floor sensitivity is evaluated by excluding respondents with baseline scores at or below 0, 3, or 5 and, more stringently, by requiring both 2010 and 2018 scores to exceed those cutoffs. Multiple imputation (m = 20) is used as a sensitivity analysis for missing parental education and household income while retaining survey strata, PSU, and longitudinal weights.

### 2.5. Alternative Longitudinal Estimands and Baseline Measurement Error

To address the possibility that the baseline-adjusted coefficient is partly a consequence of large baseline separation and measurement error, we estimate a gain score model in which the dependent variable is the observed 2010–2018 score change expressed in 2018 outcome SD units. For comparison, we first estimate an unadjusted survey-weighted gain model containing only home language. The covariate-adjusted gain model then uses the same social covariates and province indicators as Model 3 but does not condition on the baseline score itself. It therefore asks whether groups differ in observed change, whereas ANCOVA asks whether groups differ at follow-up conditional on the same observed baseline value. Neither estimand is inherently privileged; their divergence is itself informative when baseline groups are far apart (Lord, 1967).

We also conduct a classical errors-in-variables sensitivity analysis for the 2010 baseline. Wu and Li (2016) reported reliability estimates between approximately 0.85 and 0.97 across the 2010 word test forms and education groups. We therefore evaluate assumed baseline reliabilities of 0.85, 0.90, and 0.95 using the same Model 3 design matrix, province fixed effects, and 2018 longitudinal weights. Let X be the Model 3 design matrix, W the diagonal matrix of longitudinal weights, y the standardized 2018 outcome, and r the assumed reliability of the standardized 2010 baseline. Because the observed baseline is standardized to weighted variance 1, the classical error variance is 1 − r. For each r, we subtract (1 − r) ∑*_i_ w_i_* from only the baseline score diagonal element of X′WX, leave all other cross-products and X′Wy unchanged, and solve the corrected weighted normal equations for the coefficient vector. Thus, the sensitivity alters the baseline measurement error assumption while retaining all Model 3 covariates and weighting. The published form and education group reliabilities are not estimates for this analytic sample or for the pooled minority-language group. The procedure assumes classical, nondifferential measurement error; it is reported as a point estimate sensitivity and measurement error-adjusted standard errors are not reported. We do not use the 2014 score as an instrument for the 2010 baseline because 2014 performance is post-baseline and may be affected by the same language, schooling, and exposure processes that predict 2018 performance, making the exclusion restriction difficult to justify. As additional descriptive evidence, we report weighted score SDs and cross-wave correlations in the balanced panel; the latter index longitudinal stability rather than measurement reliability.

### 2.6. Three-Wave Balanced Panels and Secondary Analyses

Three-wave trajectory analyses are restricted to the same respondents with comparable scores in 2010, 2014, and 2018. The balanced word-test panel includes 14,741 respondents and the balanced mathematics panel includes 14,742. Data are placed in long form with wave treated categorically. The repeated-wave survey design uses the six 2010 sampling strata (strata_f), PSU as the first-stage cluster (psu_f), respondent as the second-stage cluster (pid_cluster), the 2018 national longitudinal weight (weight18_panel), and nested cluster identifiers; in R this corresponds to svydesign(ids = ~psu_f + pid_cluster, strata = ~strata_f, weights = ~weight18_panel, nest = TRUE). Wave-by-home-language interactions are adjusted for 2010 age, age squared, sex, urban–rural residence, ethnicity, education, parental education, household income, and province. Adjusted wave-specific marginal means are obtained from the fitted survey-weighted models. Joint interactions are evaluated with design-based Wald tests; the denominator degrees of freedom are 128 for both the word and mathematics trajectory tests. Different item forms across waves were intended to reduce direct practice from repeated items, but they do not eliminate panel conditioning, aging, administration changes, or scale comparability concerns. Because longitudinal measurement invariance has not been established, these models describe observed score trajectories rather than change in a common latent cognitive construct.

Education heterogeneity is examined by adding home language by years of education interactions to Model 3 and testing the two interaction terms jointly. For interpretability, interaction coefficients are also expressed as the change in the group contrast associated with a six-year education difference. Birth cohort interactions are used as an auxiliary sensitivity check.

The self-rated Mandarin analysis uses a weighted path model with robust maximum likelihood standard errors and PSU clustering among 10,537 respondents with a 2012 proficiency rating. The product of the coefficient linking home language to proficiency and the coefficient linking proficiency to 2018 word test performance is used only as a descriptive sequential association. It is not interpreted as causal mediation. Because this secondary analysis uses a selected subset of the primary sample, its coefficient products should not be generalized to the full *n* = 16,689 sample without a dedicated subsample comparability analysis. A 2010–2012 home language stability model provides an additional descriptive check.

### 2.7. Attrition, Analytical Software, Ethics, and Data Protection

Retention from 2010 to the 2018 analytic sample is described with a weighted logistic model using the 2010 national weight. Longitudinal weights can address selection associated with observed variables but cannot eliminate attrition related to unmeasured characteristics. Analyses were conducted in R 4.6.1, primarily using survey 4.5, emmeans 2.0.3, lavaan 0.6-21, and mice for the imputation sensitivity analysis. Primary survey models use six 2010 CFPS sampling strata and 176 PSUs with the 2018 longitudinal weight; the resulting survey design degrees of freedom are 170, and the residual design degrees of freedom are 164, 157, and 133 for Models 1–3, respectively. Repeated domain and repeated wave designs additionally nest records within respondent. Singleton handling uses options (survey.lonely.psu = “adjust”). Denominator degrees of freedom are design-based and therefore vary with the sampling structure and fitted parameters.

The CFPS protocol was approved by the Biomedical Ethics Review Committee of Peking University (IRB00001052-14010). The present analytic sample is restricted to adults interviewed in 2010, all of whom provided informed consent under the CFPS protocol. This study is a secondary analysis of de-identified data and reports only aggregate results. Reproducibility materials do not redistribute respondent-level data or geographic information below the province level.

## 3. Results

### 3.1. Descriptive Differences and Baseline Stratification

The three home language groups were already strongly stratified in observed word-test performance, education, urbanicity, and ethnicity in 2010 (Table 2). Weighted mean word scores were 20.52, 17.44, and 7.89 for Mandarin, Sinitic dialect, and pooled minority-language homes in 2010 and 20.87, 18.48, and 8.02 in 2018. The corresponding mean years of education were 8.44, 7.16, and 3.75; urban residence was 65.46%, 50.47%, and 13.14%; and ethnic-minority identification was 8.66%, 4.99%, and 94.78%. Lower-tail compression is substantial: 50.18% of the pooled minority-language category scored exactly zero in 2010, compared with 5.92% of Mandarin and 13.78% of Sinitic dialect respondents. The analogous 2018 proportions were 45.99%, 7.04%, and 11.47%. These distributions make baseline adjustment especially sensitive to how the lower end of the scale is modeled and interpreted. Table 2 reports unweighted group counts; means and standard errors use the 2018 national longitudinal weight for the *n* = 16,689 respondents nested in 176 PSUs.

### 3.2. Baseline-Adjusted Oral Character Reading Differences and Model Attenuation

The Sinitic dialect contrast was close to zero from the first baseline-adjusted model onward (Table 3). In Model 3, the estimate was −0.023 SD (95% CI [−0.077, 0.030], *p* = 0.391). This means that among respondents equated on the observed 2010 word test score and the measured covariates, the 2018 Sinitic dialect contrast was small. It does not mean that the groups had equal underlying proficiency at baseline, because neither error-free baseline measurement nor cross-language measurement equivalence has been established.

The pooled minority-language response category showed a different pattern. Model 1 yielded an estimate of −0.414 SD (95% CI [−0.546, −0.281]). Adding urban–rural residence, ethnicity, education, parental education, and household income reduced the estimate to −0.287 SD, a 30.6% reduction in absolute magnitude. Adding province indicators yielded −0.292 SD (95% CI [−0.430, −0.154], *p* < 0.001), only 0.005 SD different from Model 2. The 30.6% change is descriptive and should not be interpreted as causal mediation. Likewise, the small change after adding province indicators does not demonstrate broad geographic overlap or show that the coefficient is evenly identified across provinces. Because the home language item pools all minority-language responses, the estimate is an average residual contrast for that survey category, not a parameter for any constituent language. Table 3 reports these estimates in 2018 outcome standard deviation units. Model 1 includes the matching 2010 baseline, age, age squared, and sex; Model 2 additionally includes urban–rural residence, ethnicity, education, parental education, household income, and missingness indicators; Model 3 adds province indicators. Standard errors are clustered by PSU.

### 3.3. Descriptive Cross-Domain Comparison: Significance and Equivalence Address Different Questions

**Figure 1:**
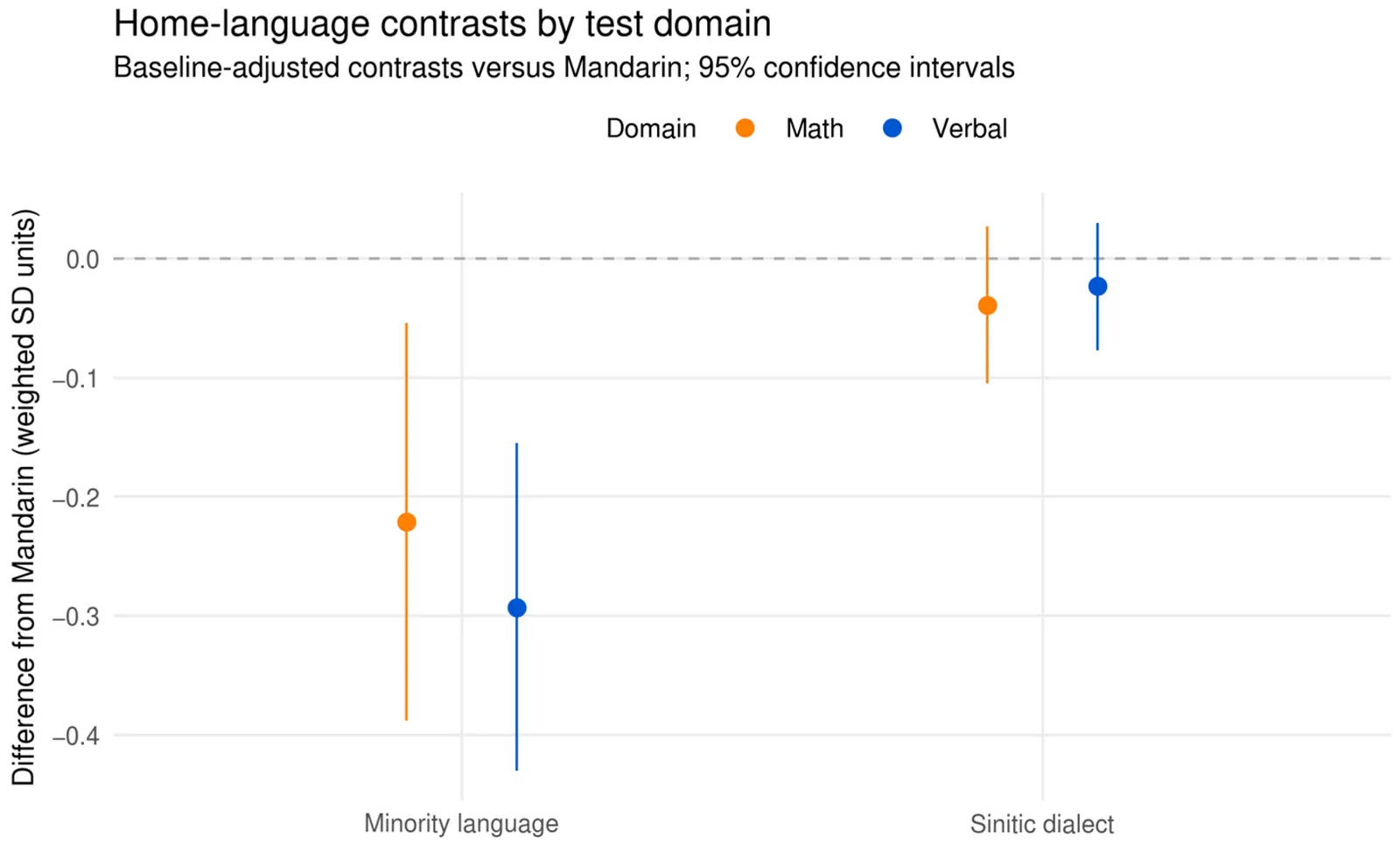
Descriptive crossdomain comparison of home language contrasts.

The joint cross-domain Wald test did not detect a systematic difference between the standardized word test and mathematics group contrasts, F(2, 95) = 0.94, *p* = 0.394 (Table 4 and Figure 1). Relative to Mandarin-speaking homes, the mathematics contrast for Sinitic dialect homes was −0.039 SD (95% CI [−0.105, 0.027]) and the word test contrast was −0.023 SD (95% CI [−0.077, 0.030]). For the pooled minority-language response category, the mathematics contrast was −0.221 SD (95% CI [−0.388, −0.054]) and the word test contrast was −0.293 SD (95% CI [−0.430, −0.155]). Figure 1 displays these adjusted contrasts relative to Mandarin-speaking homes with 95% confidence intervals; the corresponding word test minus mathematics domain differences are 0.016 SD (95% CI [−0.048, 0.080]) for Sinitic-dialect homes and −0.072 SD (95% CI [−0.230, 0.086]) for the pooled minority-language response category. These values are descriptive comparisons of standardized group contrasts; they do not identify a psychometrically pure language-specific effect. Throughout this analysis, the domain difference is defined as the word test group contrast minus the mathematics group contrast, and equivalence decisions use TOST with 90% confidence intervals.

TOST adds a bound-dependent statement to these descriptive cross-domain results. For the Sinitic-dialect group, the 90% CI for the domain difference was [−0.038, 0.069], entirely within the selected ±0.10 SD region (TOST *p* = 0.005), and also within ±0.15 SD. For the pooled minority-language category, the 90% CI was [−0.204, 0.060], not fully contained within either equivalence region (TOST *p* = 0.361 and 0.164). Thus, under the stated researcher-chosen bounds, the Sinitic dialect domain difference is sufficiently small to meet the equivalence criterion, whereas the minority-language domain difference is too imprecisely estimated to do so. Because psychometric equivalence between the two tests has not been established, these results should not be read as proof that a language-specific measurement contribution is absent.

### 3.4. Geographic Identification and Multivariate Overlap

The pooled minority-language category is geographically concentrated. Guizhou accounts for 42.69% of its longitudinal weight, Sichuan 33.51%, Guangxi 10.46%, and Yunnan 9.74%. Ethnically, the largest weighted components are Yi (30.90%), Miao (30.71%), Bouyei (14.33%), Zhuang (8.71%), and Tibetan (6.85%). Among the 646 respondents, the largest unweighted ethnicity counts are Miao (n = 191), Yi (n = 170), Zhuang (n = 95), Bouyei (n = 68), and Tibetan (n = 60). These ethnicity labels describe the sample composition but are not language identifiers. Only 10 provinces contain at least one Mandarin and one minority-language respondent, accounting for 78.74% of the minority-language-weighted sample. Only four provinces meet the more informative threshold of at least five respondents from each group: Sichuan (Mandarin n = 7, minority language n = 207), Gansu (81, 8), Liaoning (1428, 5), and Shandong (37, 5). Together these four provinces account for 34.87% of the minority-language weight. No province contains at least 10 respondents from both groups.

Within the 10 provinces containing at least one respondent from both Mandarin and minority language homes, the adjusted minority-language contrast was −0.321 SD (95% CI [−0.487, −0.154]); within the four provinces containing at least five respondents from both groups, it was −0.345 SD (95% CI [−0.522, −0.167]). Leave-one-province-out estimates ranged from −0.362 SD when Guizhou was omitted to −0.230 SD when Sichuan was omitted. Replacing the binary Han/minority ethnicity covariate with exact ethnicity code fixed effects, while retaining province fixed effects, reduced the pooled minority-language coefficient to −0.183 SD (95% CI [−0.296, −0.070]). Thus, geographic concentration does not make the conditional contrast disappear, but finer ethnic adjustment materially changes its magnitude.

The multivariate propensity analysis gives a different and more consequential diagnosis. Propensity scores are almost separated: the 1st–99th percentile overlap intersection is only 0.052–0.114, and only 49 respondents (six from minority-language homes) fall inside it. Under the original longitudinal weights, the maximum absolute standardized mean difference across propensity model covariates is 3.40; overlap weighting reduces the maximum absolute standardized mean difference to 0.011. The Kish effective sample size of the final overlap weights is only 50.3 overall, with group-specific effective sample sizes of 35.0 for Mandarin and 19.6 for minority-language homes, despite 3564 observed respondents in the two-group comparison. In the tiny propensity support intersection the minority-language estimate is 0.098 SD with very large uncertainty, whereas the overlap-weighted adjusted contrast is −0.081 SD (95% CI [−0.291, 0.129]). Accordingly, the −0.292 SD Model 3 coefficient should not be described as robust to limited multivariate overlap. It summarizes a regression-adjusted conditional contrast in a population with severe joint covariate separation. Table 5 brings the principal support, measurement, and estimand sensitivity estimates together in one place. Its coefficients are expressed in 2018 outcome SD units unless otherwise stated; the marginal support rows restrict only baseline score and education. The 49-person propensity support intersection (including six minority-language respondents) is treated only as a weak identification diagnostic, not as a stable alternative estimate, and EIV denotes the errors-in-variables sensitivity under classical baseline measurement error.

**Figure 2:**
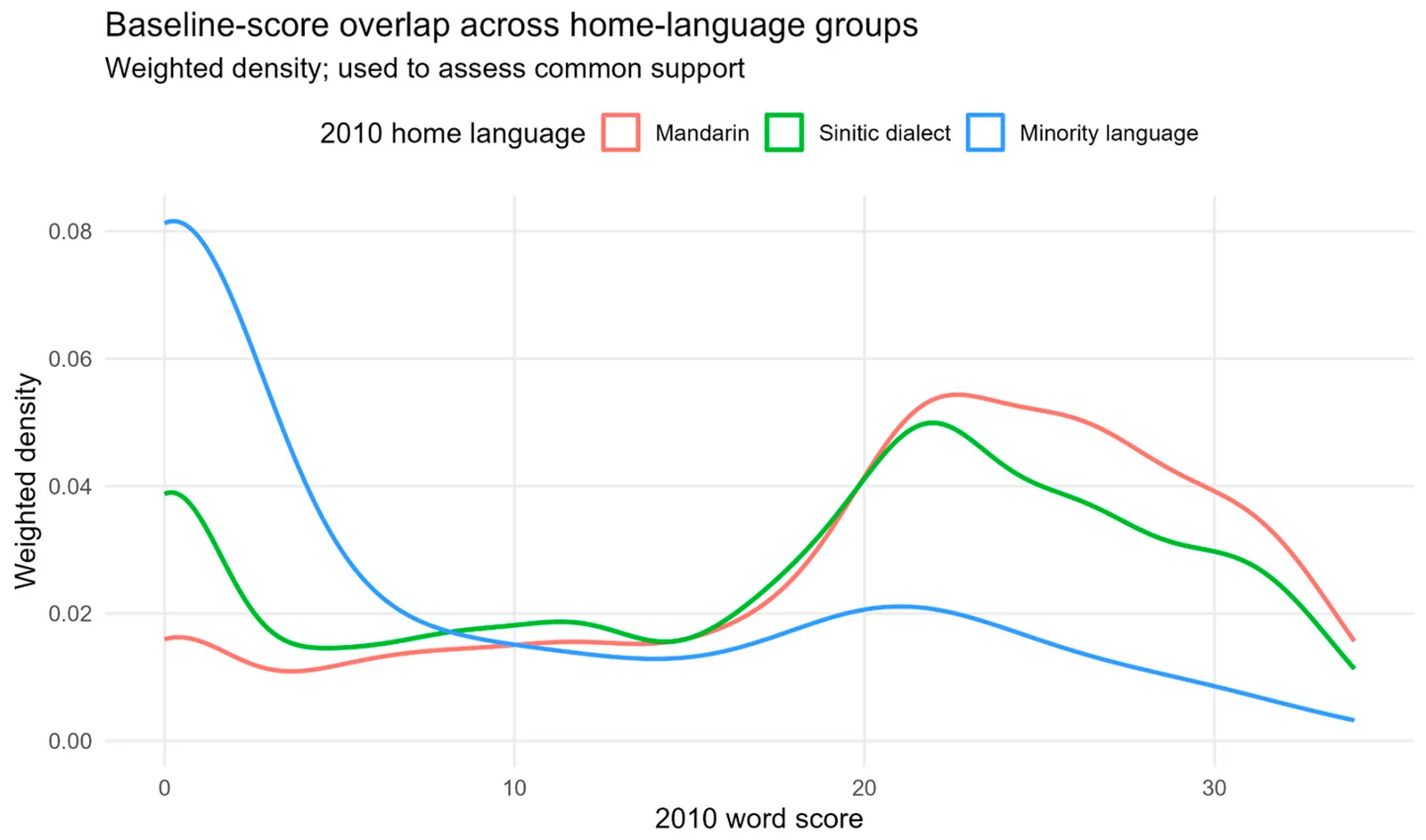
Weighted density and overlap in the 2010 word test baseline across home language groups.

**Figure 3:**
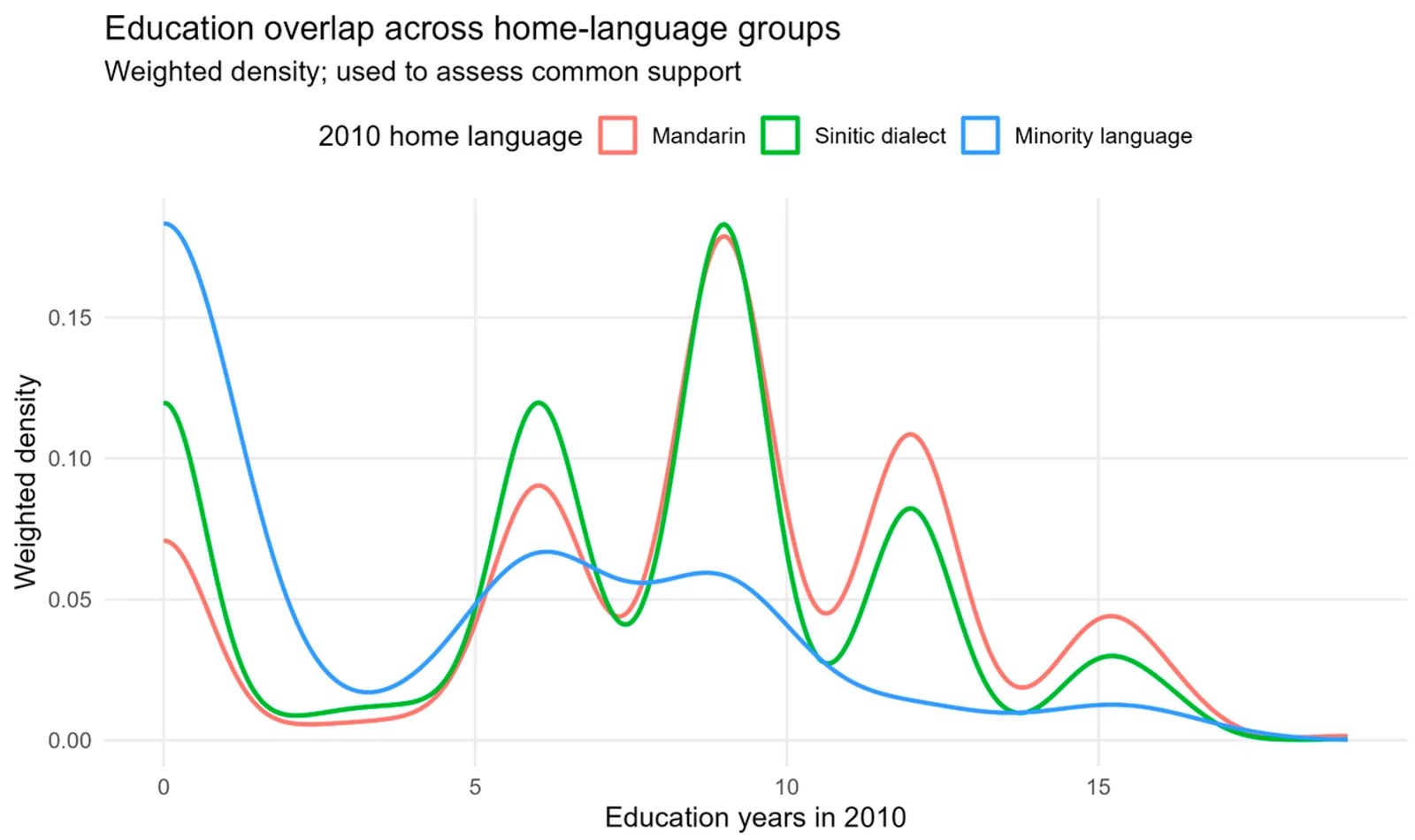
Weighted density and overlap in years of education in 2010 across home language groups.

The marginal density plots (Figure 2 and Figure 3) show lower baseline scores and education in the pooled minority-language group, but they understate the multivariate problem because the propensity score diagnostic shows near-separation once the full covariate pattern is considered. Figure 2 makes the greater concentration of the pooled minority-language category at low baseline word scores visible, while Figure 3 shows that the education distributions differ substantially even though they retain overlapping regions. The marginal plots are therefore retained as descriptive context rather than as evidence of adequate common support.

### 3.5. Gain Score and Baseline Reliability Sensitivity

The gain score analysis answers a different longitudinal question from Model 3. Raw weighted 2010–2018 word-score gains were +0.35 points for Mandarin homes, +1.04 for Sinitic dialect homes, and +0.13 for the pooled minority-language category. Without covariate adjustment, the survey-weighted gain contrasts were 0.066 SD for Sinitic dialect homes (95% CI [−0.026, 0.159]) and −0.021 SD for the pooled minority-language category (95% CI [−0.142, 0.099]). After adjustment for the Model 3 social and province covariates—but without conditioning on the baseline score—the corresponding gain contrasts were −0.035 SD for Sinitic dialect homes (95% CI [−0.124, 0.053]) and −0.180 SD for the pooled minority-language category (95% CI [−0.356, −0.005]). The latter is notably smaller in magnitude than the baseline-adjusted −0.292 SD estimate. For mathematics, the corresponding gain contrasts were −0.017 SD (95% CI [−0.101, 0.068]) and −0.129 SD (95% CI [−0.288, 0.029]). Thus, the stronger minority-language coefficient in the ANCOVA specification is partly estimand-dependent, consistent with the concern raised by Lord’s paradox.

The observed weighted SDs of the word score were 10.46 in 2010 and 10.29 in 2018 overall; within the pooled minority-language group they were 10.10 and 9.89. In the balanced panel, weighted cross-wave correlations were 0.767 for 2010–2014, 0.783 for 2014–2018, and 0.783 for 2010–2018 overall; these correlations were 0.845, 0.823, and 0.867 within the pooled minority-language group. They indicate substantial score stability but are not reliability coefficients. Using Wu and Li’s (2016) published form-level estimates as illustrative baseline reliabilities, the classical measurement-error sensitivity yielded minority-language point estimates of −0.226, −0.256, and −0.277 SD for reliabilities of 0.85, 0.90, and 0.95, respectively. The Sinitic dialect estimates remained small (−0.030 to −0.025 SD). Baseline unreliability therefore changes the magnitude of the minority-language ANCOVA coefficient but does not by itself account for it under these assumptions. The three reliability sensitivity point estimates are also included in Table 5, while the computational specification is described in Section 2.5.

Floor exclusions also attenuated the pooled minority-language contrast without eliminating it. Excluding baseline word scores at or below 0, 3, and 5 produced estimates of −0.285, −0.279, and −0.269 SD. Requiring both baseline and follow-up scores to exceed the same cutoffs produced estimates of −0.211, −0.228, and −0.240 SD, respectively. Multiple imputation of missing covariates yielded −0.293 SD (95% CI [−0.429, −0.158]), essentially matching Model 3. Taken together, these analyses indicate that lower-tail measurement and baseline conditioning matter quantitatively, but no single correction provides an error-free latent group estimate.

### 3.6. Three-Wave Balanced Panels: Persistent Stratification and Nonparallel Wave-Specific Score Patterns

The balanced three-wave word test panel includes 14,741 respondents: 2665 from Mandarin-speaking homes, 11,609 from Sinitic dialect homes, and 467 in the pooled minority-language response category. The joint home language by wave test was significant, F(4, 128) = 4.20, *p* = 0.003, indicating nonparallel patterns in the observed scores. Estimated marginal means for the Mandarin group were 16.79, 16.10, and 17.20 in 2010, 2014, and 2018; the corresponding means for the Sinitic dialect group were 16.49, 15.83, and 17.55, and those for the pooled minority-language category were 13.75, 13.52, and 14.10. The lower level of the pooled category is already present at baseline; the model does not show that a new latent ability difference emerged later.

**Figure 4:**
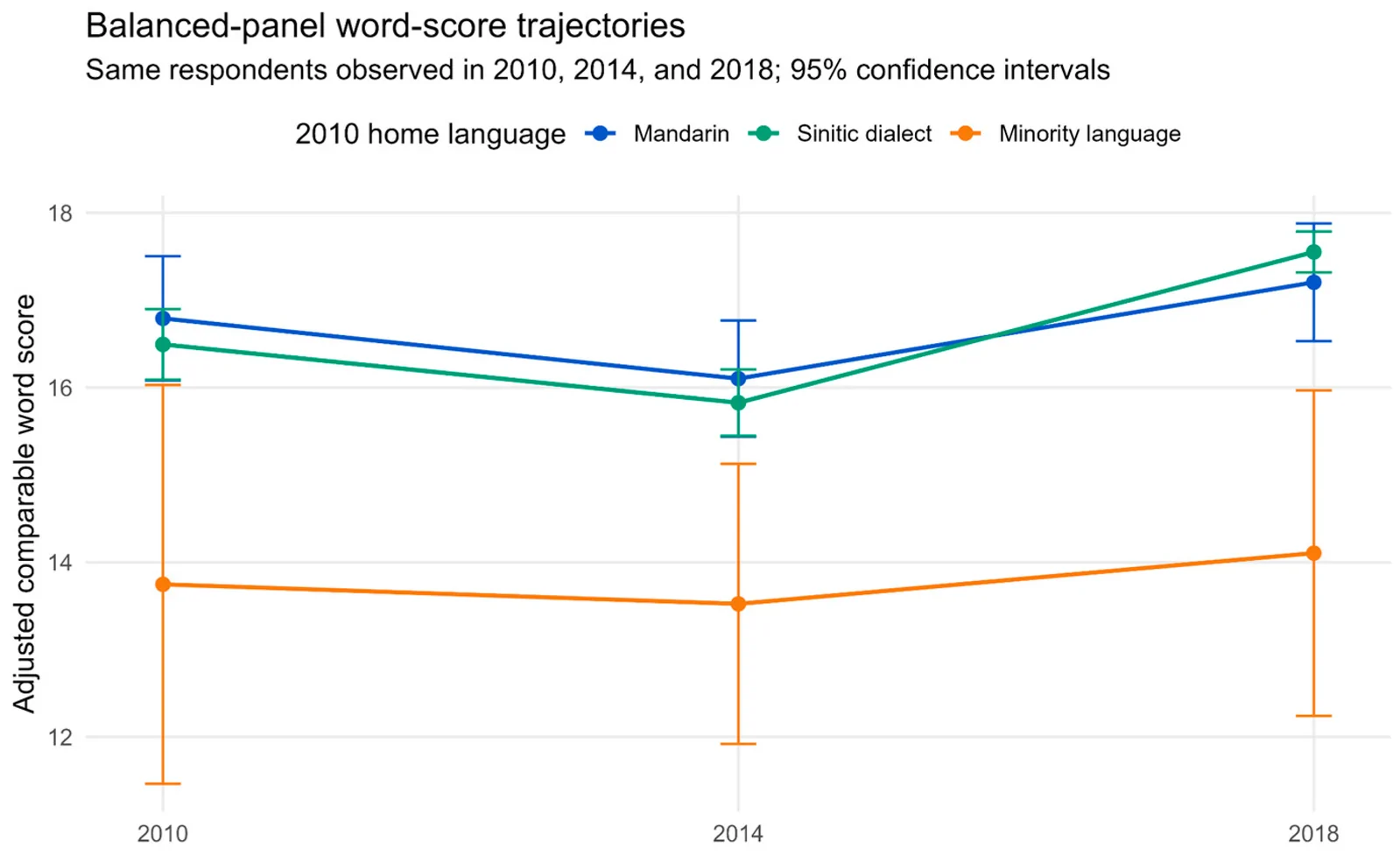
Adjusted wave-specific CFPS word test scores among respondents observed in 2010, 2014, and 2018.

Confidence intervals for individual interaction coefficients were comparatively wide, so the joint test should not be rewritten as a definitive group-specific slope difference for any single interval. The value of the balanced panel is descriptive: the same respondents contribute observed scores at all three waves. Because measurement invariance has not been established, differences across wave-specific marginal means can reflect administration, form, practice/panel conditioning, aging, scale properties, and changing exposure as well as change in the underlying abilities relevant to the task. Figure 4 plots the adjusted wave-specific marginal means for these same respondents with 95% confidence intervals. The plotted values are observed score marginal means, not estimates of an invariant latent growth curve.

The balanced mathematics panel includes 14,742 respondents, and the joint home language by wave test was also significant, F(4, 128) = 5.74, *p* < 0.001. Adjusted mathematics score differences narrowed markedly by 2018 (Mandarin = 7.51, Sinitic dialect = 7.81, minority language = 7.83). The model describes trajectories in observed wave-specific scores; wave effects may incorporate aging, scale characteristics, survey conditions, and other time-varying factors. We therefore do not interpret the decline in mathematics scores as latent cognitive decline.

**Figure 5:**
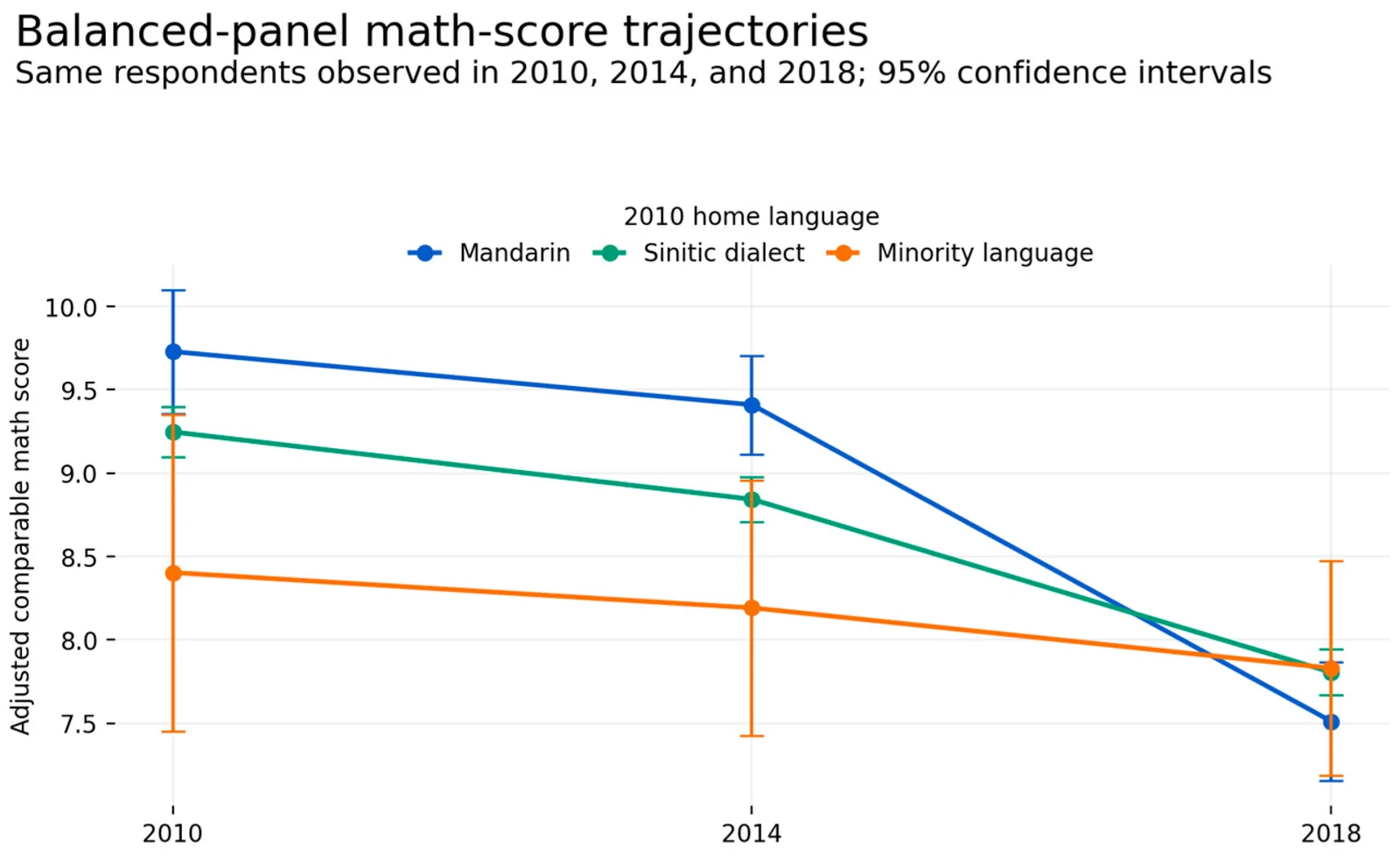
Adjusted wave-specific mathematics scores among respondents observed in 2010, 2014, and 2018.

The mathematics trajectories provide a descriptive cross-task temporal reference rather than a psychometrically matched control. The adjusted means for the Mandarin and Sinitic-dialect groups declined more from 2010 to 2018 than did those for the pooled minority-language category, and the three groups were substantially closer by 2018. Because the tasks differ in content and measurement properties and longitudinal invariance is not established, Figure 5 is used only to show that relative observed score patterns depend on the task and wave; it does not support inference about latent cognitive growth or decline. Figure 5 plots the adjusted mathematics scores for the same respondents at all three waves with 95% confidence intervals.

### 3.7. Education Heterogeneity, Self-Rated Mandarin, and Home Language Stability

Education showed a clear pattern of heterogeneity (Table 6). The joint home language by education test was F(2, 131) = 5.15, *p* = 0.0071. For each additional year of education, the Sinitic dialect contrast relative to Mandarin changed by 0.0121 SD (95% CI [0.0038, 0.0204]) and the minority-language contrast changed by 0.0202 SD (95% CI [0.0062, 0.0343]) in the direction of smaller negative differences. Expressed over a six-year education difference, these changes were 0.072 SD (95% CI [0.023, 0.122]) and 0.121 SD (95% CI [0.037, 0.206]), respectively. Birth-cohort interactions were not supported, F(2, 131) = 1.46, *p* = 0.235.

The 2012 self-rated Mandarin analysis included 10,537 respondents, but inclusion was strongly selective by home language: the weighted inclusion rate was 86.97% for Mandarin, 65.24% for Sinitic dialect, and 34.07% for the pooled minority-language category. The selected minority-language subsample also had a much higher 2010 word score mean (13.74) than the full minority-language primary sample (7.89) and represented only 1.97% of the subsample’s weighted language composition, compared with 3.96% in the primary sample. Relative to Mandarin homes, self-rated Mandarin proficiency was lower by 0.507 weighted z units for Sinitic dialect homes and 0.526 for the pooled minority-language category; the resulting descriptive coefficient products were −0.0147 SD (95% CI [−0.0249, −0.0046]) and −0.0153 SD (95% CI [−0.0267, −0.0038]). Because of the documented selection, these products are treated only as secondary associations within the observed proficiency subsample. The proficiency estimates summarized in Table 6 use longitudinal weights, robust maximum likelihood standard errors, and PSU clustering; the education-interaction joint Wald test is the F(2, 131) = 5.15, *p* = 0.0071 result reported above.

The 2010–2012 home language stability analysis provided no additional clear signal. Relative to respondents reporting stable Mandarin use, the conditional estimate was 0.062 SD for those shifting toward Mandarin, 0.050 SD for those shifting away from Mandarin, and −0.019 SD for stable non-Mandarin use; all confidence intervals included zero. Short-term category changes can reflect reporting context and changes in household composition and are not interpreted as effects of language replacement.

### 3.8. Attrition from 2010 to 2018

Retention in the 2018 analytic sample was associated with observed baseline characteristics. Relative to respondents from Mandarin-speaking homes, the odds ratio for retention was 1.42 (95% CI [1.13, 1.79]) for Sinitic dialect homes and 1.98 (95% CI [1.19, 3.31]) for the pooled minority-language response category. Age, education, urban–rural residence, missing parental education, and province were also associated with retention. The higher conditional retention of the non-Mandarin groups does not support a simple explanation based on their greater overall loss to follow-up. We do not interpret this pattern as evidence that selective attrition is absent. We do not assign the higher conditional retention to a specific substantive mechanism; residential stability, migration, fieldwork continuity, and other follow-up processes were not modeled directly. Longitudinal weights likewise cannot correct selection that depends on unmeasured characteristics.

## 4. Discussion

### 4.1. From Group Differences to the Meaning of Scores

The central contribution of this study is a change in the level at which language group score differences are interpreted. Large surveys often treat total scores as convenient summary indicators, but a group difference on an observed task does not automatically identify a difference in a broad latent ability. Validity theory instead requires an explicit chain from task performance to the intended construct (Messick, 1995; Kane, 2013). In the CFPS, that chain is constrained by oral Mandarin reading, education-dependent administration, prior observed performance, and uneven opportunities to learn. Home language is therefore used here as an ecological marker of score-forming conditions rather than as a direct label for latent cognitive ability.

This perspective also clarifies why no single longitudinal specification should carry the full interpretation. Model 3 asks whether follow-up scores differ among respondents with the same observed baseline score; the gain model asks whether observed scores change differently without conditioning on baseline. With a baseline gap of more than 12 raw points, the estimates diverge: the pooled minority-language contrast is −0.292 SD in ANCOVA, −0.021 SD in the unadjusted gain comparison, and −0.180 SD in the covariate-adjusted gain model. The −0.292 SD estimate is therefore not treated as a singular or robust effect; it is one conditional estimate whose magnitude depends on the estimand and on the population for which adequate covariate support exists. This is the setting in which Lord’s paradox is substantively relevant (Lord, 1967). Baseline measurement error further complicates the conditional interpretation, although the reliability sensitivity suggests that plausible correction does not erase the negative estimate. The appropriate conclusion is therefore not that one model is correct and the other wrong, but that the magnitude depends on the scientific question and the treatment of an error-prone, highly separated baseline.

### 4.2. Interpreting the Residual Difference Under Limited Covariate Support

The expanded diagnostics change the interpretation of the pooled minority-language coefficient more substantially than the earlier marginal support checks did. Its magnitude is stable under splines and one-dimensional score/education trimming, but the full covariate distributions have very little overlap. The propensity score intersection contains only 49 respondents, and overlap weighting reduces the adjusted contrast to −0.081 SD with a confidence interval spanning zero. Exact ethnicity code fixed effects likewise reduce it to −0.183 SD. These results do not show that the original coefficient is spurious; they show that it is a regression-adjusted summary in a population with severe multivariate separation and should not be presented as if it were identified from broadly comparable Mandarin and minority-language respondents. These diagnostics make the support dependence of the estimate central to its interpretation: the overlap-weighted result, rather than the Model 3 value alone, shows how sharply the estimated magnitude changes when comparison is concentrated in regions of covariate support.

The aggregation of the exposure measure compounds this identification problem. The pooled category is dominated by Yi and Miao respondents and is concentrated in Guizhou and Sichuan, while the home language item itself does not identify the specific language used. Geographic restriction and leave-one-province-out analyses show that no single province alone generates the negative estimate, but only four provinces contain at least five Mandarin and five minority language respondents. The coefficient therefore cannot be generalized to individual minority languages, and ethnicity-specific or province-specific composition should be considered part of its substantive definition rather than a nuisance detail.

### 4.3. Cross-Domain Evidence Changes What Counts as a Language Load Effect

The cross-domain analysis provides a descriptive constraint on interpretation, not a clean identification strategy for language-specific test bias. If the oral Mandarin requirement were the sole or dominant source of the observed group contrast, a larger contrast on the word task than on mathematics would be suggestive. But mathematics is itself shaped by schooling, linguistic comprehension, symbolic familiarity, curriculum exposure, and psychometric properties. The two standardized contrasts can therefore be compared descriptively, while the difference between them cannot be treated as a pure language load parameter.

The significance and equivalence analyses answer different statistical questions within that limited descriptive comparison. A nonsignificant domain interaction means that a difference between the two standardized group contrasts was not detected; TOST asks whether the domain difference is contained within a researcher-selected interval. The ±0.10 and ±0.15 SD regions are not externally validated CFPS thresholds. For the Sinitic dialect group, the estimated domain difference is small under these bounds. For the pooled minority-language response category, the interval remains compatible both with a small difference and with a moderately larger disadvantage on the word task. Neither result establishes measurement invariance, equal reliability, or absence of language-related construct-irrelevant variance.

The close cross-domain pattern for Sinitic dialect homes is informative in this respect. Respondents in Mandarin and Sinitic dialect homes share the Chinese writing system and largely participate in a Mandarin-medium school environment, and the difference in home spoken code does not translate into a detectable additional penalty on character recognition. This finding is inconsistent with a simple model in which any non-Mandarin home language automatically creates test bias. The minority-language category remains more ambiguous: the available precision neither establishes practical cross-domain equivalence nor demonstrates a stable extra character recognition component. The appropriate interpretation is therefore to keep several mechanisms in play rather than force the result into a binary choice between a pure language effect and a completely domain-general effect.

This inferential stance is consistent with current work on fair cognitive assessment. Increasingly, language fairness is established through test design, normative coverage, differential item functioning, and direct measurement of language experience rather than through group means alone (Holden & Tanenbaum, 2023; Ortiz & Cehelyk, 2024; Kijamet et al., 2025). For large social surveys that lack item-level data, a cross-domain contrast combined with equivalence testing can provide a relatively low-cost constraint on interpretation, although it cannot replace formal measurement invariance or DIF analyses.

### 4.4. Temporal Trajectories Suggest Cumulative and Task-Dependent Opportunity Structures

The balanced panels add a temporal description of observed scores rather than a latent growth analysis. Word test separation is already present at baseline and remains visible at later waves, whereas mathematics displays a different relative pattern. Cross-wave word score correlations around 0.77–0.78 overall indicate substantial longitudinal stability, but they do not establish reliability or measurement invariance. Different forms reduce direct item repetition; they do not eliminate panel conditioning, aging, administration changes, or scale comparability concerns. The wave models therefore provide context for persistence on the observed scales, not evidence of common latent growth trajectories.

The observed task-dependent pattern is consistent with several cumulative experience accounts, including differences in formal schooling, exposure to written Mandarin, language use, and test-taking experience (Bourdieu, 1991; Huang et al., 2015). It is not uniquely diagnostic of any one account. Work on cohort changes in Chinese cognitive scores likewise shows that observed totals are shaped by educational expansion and population history (Y. Qi & Xiong, 2023). Repeated survey measurement can therefore reveal stable or changing relative positions on particular observed scales without, by itself, establishing latent cognitive growth or decline.

### 4.5. A Constrained Validity Argument for Large-Scale Survey Scores

The methodological contribution of the study is best described as a constrained validity argument in which stronger interpretations survive only if they remain plausible across distinct estimands and support conditions. Baseline adjustment and gain scores answer different longitudinal questions; measurement error sensitivity tests dependence on an error-prone baseline; geographic and propensity diagnostics test whether adjusted groups are actually comparable; floor exclusions probe lower-tail compression; cross-domain comparisons limit language-specific interpretations; and three-wave models describe persistence without assuming latent invariance. In the revised analysis, these constraints do not all point to the same numerical coefficient. The disagreement across specifications is itself the substantive result: the minority-language contrast varies with estimand, measurement assumptions, and covariate support, so the −0.29 SD Model 3 estimate should not be read as the study’s single robust effect.

This approach follows the logic of argument-based validation. Kane (2013) emphasized that validation requires testing the chain of inferences that links observed performance to the intended interpretation. In secondary analyses of large surveys, item-level responses, experimental language manipulations, and complete psychometric information are often unavailable. Multiple independent constraints can still reduce inferential leaps. This strategy is not a substitute for modern psychometrics, but it strengthens the inferential discipline of cognitive score research in social surveys and shifts the meaning of robustness from repeated statistical significance to the survival of an interpretation after plausible alternatives have been challenged.

### 4.6. Implications for Intelligence Assessment and Multilingual Measurement

For intelligence and cognitive assessment, the most direct implication concerns the scale of the construct claim. CHC theory places language development, lexical knowledge, and acquired knowledge within the broad comprehension knowledge domain (Schneider & McGrew, 2018; McGrew et al., 2023), but a single oral character reading score does not automatically provide broad-domain coverage. The present study does not compare the CFPS word score with a validated broad measure of crystallized intelligence and therefore does not demonstrate that the CFPS test is an invalid Gc measure. The narrower conclusion is that the available evidence is insufficient to justify treating this language-dependent, education-adaptive survey score as a direct measure of broad crystallized intelligence across home language groups without additional validity evidence.

Future large-scale surveys that aim to distinguish language experience, educational opportunity, and cognitive constructs will require more mechanism-proximal data. Particularly useful measures would include the language actually used for each cognitive module, objective and self-rated proficiency, literacy in each language, frequency of use, age of acquisition, school language of instruction, interviewer language, and migration history. Item-level responses would permit multigroup item response models, DIF analyses, and measurement invariance tests. Stronger designs could also administer different language versions to the same respondents or randomize the language of instructions, allowing for a more direct test of task language mechanisms. Planned oversampling of minority-language communities would further reduce the need to compress heterogeneous language ecologies into a single residual category.

## 5. Limitations

Five limits should govern interpretation. First, the exposure is a broad survey response rather than a specific language identifier; its composition is dominated by several ethnic and provincial clusters, and only limited within-province Mandarin–minority comparisons are available. Second, multivariate covariate overlap is extremely weak. The overlap-weighted result is far smaller than Model 3, so regression adjustment cannot be treated as if it recreated a broadly comparable population. Third, the word score is produced by education-dependent entry, oral Mandarin responses, and a three-error stopping rule. Floor exclusions attenuate the minority-language coefficient, item-level stopping distributions are unavailable, and measurement invariance across language groups is not established. Fourth, baseline adjustment is sensitive to the scientific estimand and to measurement error: ANCOVA and gain score estimates differ materially, while classical reliability correction changes but does not remove the negative coefficient. Fifth, the cross-domain and longitudinal analyses remain descriptive because the word and mathematics tests differ psychometrically and longitudinal invariance, practice effects, and full scale comparability are unresolved. The selected 2012 proficiency subsample also underrepresents minority-language respondents.

These limitations point to a more specific next step than adding more covariates. Stronger work would oversample language communities, record the specific language used, measure objective proficiency and literacy in each language, document school and interviewer language, and retain item-level responses and stopping points. Those data would permit multigroup item response models, DIF and measurement invariance analyses, and estimands defined within genuine regions of common support. In the present CFPS data, the strongest defensible contribution is to show how the interpretation changes when the same observed score difference is subjected to these constraints.

## 6. Conclusions

Language group differences in cognitive test totals acquire psychological meaning only through a defensible chain from task performance to construct interpretation. For Sinitic dialect homes, the adjusted word test contrast is consistently small. For the pooled minority-language response category, the central finding is not a single −0.29 SD effect but the dependence of the estimated contrast on the estimand and the support population. The conventional baseline-adjusted estimate is about −0.29 SD, whereas exact ethnicity adjustment yields −0.18 SD, the unadjusted gain contrast is −0.02 SD, the covariate-adjusted gain score model yields −0.18 SD and overlap weighting yields −0.08 SD with substantial uncertainty. Plausible correction for baseline unreliability and floor exclusions attenuate the estimate to varying degrees without producing an error-free latent comparison. The category is also geographically and ethnically concentrated and cannot be disaggregated by specific language from the home language item. The mathematics comparison and three-wave trajectories add descriptive constraints but do not establish measurement equivalence. The revised evidence therefore supports neither a fixed language group ranking in broad crystallized intelligence nor a uniform language-specific test effect. It supports a narrower conclusion: this language-dependent survey score should be interpreted conditionally, with explicit attention to baseline definition, measurement error, floor behavior, and the population in which comparisons are actually supported.

## Figures and Tables

**Table 1:** Sample flow and language group retention from 2010 to the final analytic sample.

Step	Total *n*	Mandarin n (%)	Sinitic Dialect n (%)	Minority Language n (%)
Focal 2010 home-language category	32,725	6201 (100.0)	25,292 (100.0)	1232 (100.0)
Valid 2010 word score	32,693	6195 (99.9)	25,266 (99.9)	1232 (100.0)
Linked to 2018 person file	21,596	3816 (61.5)	16,936 (67.0)	844 (68.5)
Valid 2018 word score	16,729	2922 (47.1)	13,158 (52.0)	649 (52.7)
Positive 2018 longitudinal weight	16,729	2922 (47.1)	13,158 (52.0)	649 (52.7)
Complete design/core covariates	16,689	2918 (47.1)	13,125 (51.9)	646 (52.4)

**Table 2:** Weighted characteristics of the primary sample by 2010 home language.

Characteristic	Mandarin	Sinitic Dialect	Minority Language
Unweighted *n*	2918	13,125	646
2010 word score, mean (SE)	20.52 (0.67)	17.44 (0.40)	7.89 (1.78)
2018 word score, mean (SE)	20.87 (0.58)	18.48 (0.38)	8.02 (1.54)
Age in 2010, years (SE)	44.46 (0.58)	44.94 (0.38)	43.31 (1.72)
Education years in 2010 (SE)	8.44 (0.32)	7.16 (0.17)	3.75 (0.77)
Male, % (SE)	45.90 (0.93)	47.63 (0.48)	51.28 (3.08)
Urban, % (SE)	65.46 (7.39)	50.47 (3.26)	13.14 (5.00)
Ethnic minority, % (SE)	8.66 (4.00)	4.99 (1.43)	94.78 (2.46)
2010 score = 0, weighted %	5.92	13.78	50.18

**Table 3:** Conditional home language differences in 2018 Mandarin oral character reading performance.

Home Language Contrast	Model 1 Estimate [95% CI]	Model 2 Estimate [95% CI]	Model 3 Estimate [95% CI]
Sinitic dialect–Mandarin	−0.023 [−0.099, 0.054]	0.006 [−0.073, 0.086]	−0.023 [−0.077, 0.030]
Minority language–Mandarin	−0.414 [−0.546, −0.281]	−0.287 [−0.439, −0.135]	−0.292 [−0.430, −0.154]

**Table 4:** Cross-domain contrasts and equivalence tests for character recognition versus mathematics.

Language Group	Word Test vs. Mandarin	Mathematics vs. Mandarin	Domain Difference (90% CI)	±0.10 SD	±0.15 SD
Sinitic dialect	−0.023 [−0.077, 0.030]	−0.039 [−0.105, 0.027]	0.016 [−0.038, 0.069]	Equivalent, *p* = 0.005	Equivalent, *p* < 0.001
Minority language	−0.293 [−0.430, −0.155]	−0.221 [−0.388, −0.054]	−0.072 [−0.204, 0.060]	Equivalence not established, *p* = 0.361	Equivalence not established, *p* = 0.164

**Table 5:** Sensitivity of the pooled minority-language contrast across support, measurement, and estimand specifications.

Specification	Sinitic Dialect–Mandarin	Minority Language–Mandarin
Primary Model 3	−0.023 [−0.077, 0.030]	−0.292 [−0.430, −0.154]
Exact ethnicity code FE + province FE	−0.006 [−0.046, 0.034]	−0.183 [−0.296, −0.070]
1st–99th marginal support	−0.026 [−0.080, 0.028]	−0.290 [−0.426, −0.154]
5th–95th marginal support	−0.040 [−0.116, 0.037]	−0.315 [−0.476, −0.153]
Baseline natural spline, df = 4	−0.038 [−0.090, 0.014]	−0.273 [−0.398, −0.149]
Baseline + education splines	−0.044 [−0.093, 0.006]	−0.272 [−0.391, −0.153]
Exclude baseline score ≤ 0	−0.028 [−0.070, 0.014]	−0.285 [−0.427, −0.143]
Exclude baseline score ≤ 3	−0.027 [−0.068, 0.014]	−0.279 [−0.424, −0.135]
Exclude baseline score ≤ 5	−0.022 [−0.063, 0.019]	−0.269 [−0.407, −0.132]
Retain scores > 0 at both waves	−0.036 [−0.077, 0.005]	−0.211 [−0.358, −0.065]
Unadjusted gain score contrast	0.066 [−0.026, 0.159]	−0.021 [−0.142, 0.099]
Covariate-adjusted gain score model	−0.035 [−0.124, 0.053]	−0.180 [−0.356, −0.005]
Overlap-weighted model	—	−0.081 [−0.291, 0.129]
Propensity 1st–99th intersection (*n* = 49)	—	0.098 [very imprecise]
Multiple imputation (m = 20)	−0.023 [−0.076, 0.029]	−0.293 [−0.429, −0.158]
EIV reliability = 0.85	−0.030	−0.226
EIV reliability = 0.90	−0.027	−0.256
EIV reliability = 0.95	−0.025	−0.277

**Table 6:** Secondary analyses of self-rated Mandarin proficiency and education heterogeneity.

Analysis	Parameter	Estimate [95% CI]	Six-Year Scale	*p* Value
Sequential association via self-rated Mandarin	Sinitic dialect–Mandarin	−0.0147 [−0.0249, −0.0046]	—	0.004
Sequential association via self-rated Mandarin	Minority language–Mandarin	−0.0153 [−0.0267, −0.0038]	—	0.009
Education heterogeneity	Sinitic dialect × years of education	0.0121 [0.0038, 0.0204]	0.072 [0.023, 0.122]	0.005
Education heterogeneity	Minority language × years of education	0.0202 [0.0062, 0.0343]	0.121 [0.037, 0.206]	0.005

## Data Availability

The data analyzed in this study were obtained from the China Family Panel Studies (CFPS), administered by the Institute of Social Science Survey, Peking University. The CFPS public-use data are available to researchers through the official CFPS data platform following registration and approval in accordance with the data provider’s access procedures. The authors are not permitted to redistribute the CFPS respondent-level data. Researchers may apply for and download the data directly from the CFPS data provider. The authors impose no additional restrictions beyond those established by the CFPS data provider.
